# Evaluation of major depression in a routine clinical assessment

**DOI:** 10.1186/1758-5996-2-9

**Published:** 2010-01-28

**Authors:** Marco André Urbach Mezzasalma

**Affiliations:** 1 Laboratory of Panic and Respiration, Institute of Psychiatry, Federal University of Rio de Janeiro (UFRJ), INCT Translacional Medicine, Brazil

## Abstract

**Background:**

Major depression is a disorder that significantly worsens a patient's morbidity and mortality. The association of depression and diabetes is well documented and has clinical impact in diabetes treatment's outcome. Patients usually aren't evaluated initially by a psychiatrist, so it is important that non-psychiatrists learn to evaluate major depression and its impact.

**Conclusions:**

Major depression can and should be evaluated on a routine clinical assessment. Depression's impact on the patients' quality of life, productivity and social interactions is well documented. The initial diagnosis of depression should lead to its prompt treatment, and it has to be emphasized that the incorrect treatment can lead to worsening of the condition, relapses, recurrences or even chronification of major depression.

## Introduction

In this approach for non-psychiatrists, we have to begin with the definition of sadness. According to the The American Heritage Dictionary of the English Language, Fourth Edition, sadness (or a sad state) is: 1) Affected or characterized by sorrow or unhappiness; 2) Expressive of sorrow or unhappiness; 3) Causing sorrow or gloom, depressing; 4) Deplorable, sorry; 5) Dark-hued, somber.

Depression is a syndrome whose signs and symptoms remain for a period of weeks or months. There is also an important impairment of the usual functions of the individual, i.e., people change their behavior regarding work, personal relationships and themselves[1].

The most recent diagnostic criteria adopted by the American Association of Psychiatry[2] are described in Appendix 1.

It is important to emphasize, for those who are not used to dealing with depression on a regular basis, that depression is not just an increase in the intensity or a different quality of sadness, but rather a state that will develop into other complications, such as the ones mentioned in Appendix 1. The presence of depression's symptoms is not enough (A), and we need to evaluate the functional impact on the individual's routine (B), the exclusion of triggering factors such as the use of depressive substances or clinical conditions (C) and discard the occurrence of a Mourning situation (D).

The prevalence of a major episode of depression throughout life, according to large studies conducted in the USA and in Europe varied from 5 to 17%[3-8]. In our country, considering that there are 180 million Brazilians according to the latest census by IBGE (Brazilian Institute of Geography and Statistics), estimates show that 30 million Brazilians will go through a depressive episode. It's important to emphasize that general practitioners and neurologists are usually the types of physicians initially visited.

The importance of discussing the diagnosis of depression for endocrinologists is the frequent association of depression and diabetes. Several studies have proven such association[9]. Depression has already been associated with hypoglycemia[10], complications related to diabetes[11], as well as the perception of functional limitations caused by diabetes[12]. A recent Brazilian study assessing type 2 diabetic patients found not only elevated prevalence of depression, but also a positive correlation between the severity of the depressive condition and the severity of symmetrical distal diabetic polyneuropathy presented by patients[13].

It has already been demonstrated that the levels of depression in diabetic patients are at least twice as high as people without chronic diseases[9], the prevalence of depression in diabetic patients may be ≥ 40%[14], and the comorbidity of depression and diabetes may extend the depressive episode or favor recurrences[15].

Over the last decade depression started being studied as one of the etiological factors of diabetes[16], and some studies proved that depression is a predictor of the later occurrence of diabetes[17,18]. In the general population, depression is responsible for worsening morbidity and mortality, even in the absence of diabetes[19]. A study showed that depression would be an important risk factor for microvascular disease, macrovascular disease, loss of autonomy and even mortality due to diabetes[20]. From this study, the existence of a synergic effect between depression and diabetes was postulated, i.e., the effect of both conditions together would be higher than the sum of the effects of each[21].

Considering the implications of comorbidity between depression and diabetes, it is indispensable to assess its occurrence carefully in diabetic patients. The clinical implications of depressive conditions are evident, and the absence of treatment or improper treatment increase the risk to a magnitude similar to the risk presented by untreated or improperly treated increased blood pressure[22].

Unfortunately, studies demonstrate that depression is underdiagnosed, particularly in diabetic patients. It is estimated that only one third of depressed diabetic patients are properly diagnosed[23]. It is important to emphasize that proper diagnosis and treatment may lead to the remission of the depressive condition, and also to a decrease in the morbidity and mortality risks.

Nowadays, there are over 45 different drugs available for treating depression, allowing for different options and interactions. Unfortunately, the most prescribed drugs are not antidepressants, as patients usually get to the non-specialist's office and receive a prescription of benzodiazepinic drugs so that they "stop bothering the physician". There is a wide range of therapeutical possibilities and this allows us to treat the person, not the disease. Depression occurs twice as much in women as in men, and there might be a diagnostic confusion around different conditions, from premenstrual dysphoria (former PMS) to premenopausal syndrome and general anxiety syndromes. We cannot call sadness depression, nor can we treat sadness.

We have several hypotheses for the etiology of depression:

1) biological factors, having antidepressants and their action on the brain neurotransmission, as well as hormonal factors implying the depression genesis.

2) genetic factors, as studies point to risks two or three times higher in first-degree family members with depression. However, studies with homozygotic twins have not proven a concordance of 100%, which allows us to conclude that psychosocial factors are important[24-27].

3) psychosocial factors and the lifestyle adopted by the person are among the determining factors: leisure, physical activities versus inactivity, smoking, diet, time spent with the family and time for their individual hobbies.

The natural progression of depression usually is its appearance in the third decade of life, and it may occur at any age. Depressive disorders may start from 5 to 60 years of age. The symptoms develop over weeks and remain for months until they evolve to the impairment of one's functions, which initially goes unnoticed by the person, or by their family. We have obtained increasing evidence that the conditions which do not receive proper treatment tend to become chronic.

Nowadays, we consider depression a disease of intermittent chronicity, which results in high economic cost to the Health System. There is a concept created by the University of Harvard along with World Health Organization (WHO) called "Disability Adjusted Life Year" (DALY)[28], based on the concept that the differences in ranking disability or death should be by age and gender, not income, culture, location or social class, in an attempt to standardize morbimortality data globally since everyone in the world has the right to have the best possible life expectancy. To calculate DALY, the criteria considered were years of life lost by death, and years of life lost due to disability by diseases or traumas. Data from the World Health Organization[29] for the year of 2004 are found in Table 1. Depression is not only a very frequent disease, but also a cause of strong disability to people.

**Table 1:** Leading Causes of Burden of Disease in the World, 2004[29]

DALYs	%
1. Lower respiratory infections	6,2

2. Diarrhoeal diseases	4,8

3. Depression	4,3

4. Ischaemic heart disease	4,1

5. HIV/AIDS	3,8

6. Cerebrovascular disease	3,1

7. Prematurity, low birth weight	2,9

8. Birth asphyxia, birth trauma	2,7

9. Road traffic accidents	2,7

10. Neonatal infections and other	2,7

The estimated cost of depression in the USA is approximately 81.5 billion dollars per year, with an annual loss of productivity of 32 billion dollars[30]. It has been observed that the increase in annual productivity after treating the employee's depression is around U$ 1.800[31]. There are no cost data about depression for the Brazilian population. Generally, depressed patients use the health system and present more medical and psychiatric comorbidities. Depression is the greatest cause of disability from 15 to 44 years of age, according to WHO data for the year 2004[32].

Risk Factors for Major Depression:

1. Gender: the female gender has two times more depression than the male gender;

2. Age: from 20 to 40 years old;

3. Social and economic factors, such as unemployment and underemployment;

4. Psychosocial factors such as marital state, i.e., divorced, widows, widowers or single individuals have higher risk of developing a depressive condition. Living in urban areas, negative life events such as the loss of cherished ones, or common daily news may be important stress factors.

It is important to remember the possible differential diagnoses, which are general and neurological medical conditions, pharmacological agents, other mood disorders, adjustment disorder with depressed mood, mainly in children and teenagers.

In order to understand why depression and anxiety coexist in over 70% of the cases, if we analyze the symptoms presented there is a huge intersection[33]. Symptoms exclusive to depression are depressed mood, anedonia (losing pleasure in previously pleasant activities), appetite changes, libido changes, decrease in motivation and suicidal ideation. Symptoms exclusive to anxiety are hyperexcitability, compulsive rituals and agoraphobia (fear of crowds or open spaces and even of "feeling bad"). There are many symptoms common to both: sensation of fear, apprehension, chronic pains, gastrointestinal symptoms, psychomotor excitement, difficulty of concentration, change in the sleep pattern, fatigue and energy levels, so comorbidities of depression and anxiety conditions are very common.

### Treatment

The choice of antidepressants is based on the patient profile as well as liver, kidney and cardiovascular safety, as different drugs have different side effects and it is best to personalize the treatment. The analysis of drug interactions and side effects of other medications is very important, as well as the assessment of other pathologies the patient presents, and the action mechanism of the chosen antidepressant, the drug cost and tolerability. Generally, antidepressants have very similar efficacy. In Table 2 we have a list with the main antidepressant groups available in Brazil.

**Table 2:** Main antidepressants available in Brazil

Drug Class and Active Ingredient	Usual average dose for adults(mg/day)	Sedation	Anticholinergic Action	Orthostatic Hypotension
Tricyclic Antidepressants (TCAs)^A^				
Imipramine	150-200	Moderate	Moderate	High
Amitriptyline	150-200	High	Very high	Moderate
Nortriptyline	75-100	Moderate	Moderate	Lowest of TCAs
Clomipramine	150-200	High	High	Low

Tetracyclic Antidepressants ^A^				
Maprotiline	150-200	Moderate	Moderate	Low

Monoaminoxidase Inhibitors (MAOIs)				
Tranilcipromine	30	-	Very low	High

Selective serotonin reuptake inhibitors (SSRIs)				
Fluoxetine	20-60	Very low	None	Very low
Paroxetine	20-40	Low	Low	None
Sertraline	100-150	Low	None	None
Citalopram	20-40	Low	None	None
Fluvoxamine	50-150	Low	None	None
Escitalopram	10-20	Low	None	None

Serotonin-norepinephrine reuptake inhibitors (SNRIs)				
Venlafaxine	75-225	Low	None	Very low^B^
Duloxetine	60	Low	Very low	Very low

Serotonin-norepinephrine-dopamine-reuptake-inhibitors (SNDRI)				
Bupropion^C^	300	Low	Very low	Very low

Norepinephrine reuptake inhibitor (NRI);				
Reboxetine	8-10	Very low	Very low	Very low

Noradrenergic and specific serotonergic antidepressant (NaSSA).				
Mirtazapine	30-45	High	Moderate	Low

Serotonin reuptake inhibitors and serotinin antagonist (SARI)				
Trazodone^D^	150-400	High	Very low	Moderate

^A ^- All cyclic antidepressants have elevated arrhythmogenic potential;

^B ^- Venlafaxin causes dose-dependant blood pressure increase in some individuals;

^C ^- Bupropion reduces convulsive threshold significantly, and it must be avoided in patients with history of syncopes and seizures;

^D ^- Trazodone is associated with cardiac arrhythmia and priapism

**Figure 1:**
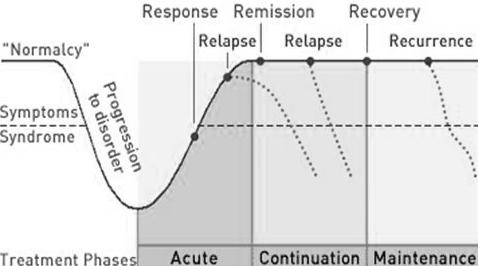
**Kupfer Curve**.

Regarding the evolution of the treatment, we have the Kupfer curve[34], which demonstrates how a depressed individual evolves over time. It can be seen in Figure 1.

An individual was normal and asymptomatic, but starts presenting symptoms, evolving to a depressive condition, with a depressive syndrome. In this acute phase, the treatment starts and a response is observed; however, such response might stop and symptoms might keep occurring - this is when a chronic condition is installed. This condition is very difficult to be solved.

The treatment aims to bring the individual back to "normalcy", i.e., leading the patient to complete recovery from symptoms by reaching remission. Another problem that might be encountered is when there is an improvement of 80 to 90% but the patient still presents symptoms, and as such they would not be completely recovered; this must also be avoided.

Once remission is reached, a continuation treatment must be made which lasts approximately for six months after the first depressive episode, and from one to two years in case of multiple episodes, as there is a high risk of relapse even during the treatment with regular medication use. The risk of relapse in the first six months, if treatment is interrupted, is approximately 80%. As previously described, the impact of depression is high from the economic point of view, which is objectively measurable, as well as the subjective point of view, and it must be avoided.

After this continuation period, the individual may be considered recovered to go on with the maintenance stage. Even then there might be later recurrence, i.e., after three months, an asymptomatic individual may present a depressive episode again, so it is considered another episode of depression, not the same.

It is recommended to start the treatment always with low doses and increase them progressively in order to minimize side effects and allow the patient to adjust to the treatment. The ideal action is to keep the full dose of antidepressants for all stages of the treatment, as long as it is well tolerated by the patient.

Regarding psychotherapy, we have three types whose efficacy is documented in literature: 1) cognitive therapy; 2) behavioral therapy; and 3) interpersonal therapy. Psychotherapy must be always integrated to psychiatric follow-up and with other treatments.

Complementary treatments such as the practice of sports, yoga, relaxation techniques and meditation, have their efficacy documented in clinical studies revealing an improvement in depressive conditions after a change in lifestyle to include the practice of physical activities, even studies with yoga techniques.

In conclusion, we must remember that depression is a public health issue all over the world, causing severe social and occupational damage to patients, and it is one of the main causes of suicide worldwide.

To answer the question "Can depression be diagnosed in a routine clinical evaluation?" The answer is: depression can and must be diagnosed in a routine clinical evaluation, because that is what we, psychiatrists, do: diagnose depression in routine visits to our practice.

In order to track depression at the endocrinologist's office, it is important to assess the patient's mood: both the presence of unmotivated sadness and the loss of pleasure in previously pleasant activities, as well as changes in vegetative functions: sleep, appetite and libido. Changes in these functions do not only allow the diagnosis of depression, but they also allow us to assess its evolution, relapse and recurrence.

## Appendix 1: Diagnostic Criteria for Major Depressive Episode, adapted from DSM-IV-TR

A. **At least five of the symptoms below must be present during the same two-week period, almost every day, representing a change from the previous function standard of the individual. One of the symptoms must be mandatorily number (1) or (2) as follows:**

1. ***depressed humor most of the day;***

2. ***accentuated decrease in interest or pleasure in all or almost all activities most of the day;***

3. significant weight loss or gain while not on a diet (e.g., more than 5% of body weight in 1 month), or decrease or increase in appetite;

4. insomnia or hypersomnia;

5. excitement or psychomotor retardation;

6. fatigue or energy loss;

7. feeling of uselessness or excessive or improper guilt;

8. diminished ability of thinking or concentrating, or indecision;

9. recurrent thoughts of death (not only fear of dying), recurrent suicidal ideation without a specific plan, suicide attempt or specific plan to commit suicide.

B. **The symptoms cause clinically significant suffering or damage in social or occupational function, or in other important areas in the individual's life.**

C. **The symptoms are not due to direct physiological effects of a substance (e.g., drug abuse or medication) or a general medical condition (e.g.: hypothyroidism).**

D. **The symptoms are not better explained by Mourning (i.e., followed by the loss of loved ones, symptoms remain for more than 2 months), or are characterized by accentuated functional damage, morbid concern about devaluation, suicidal ideation, psychotic symptoms or psychomotor retardation.**
